# Organization and characteristics of the major histocompatibility complex class II region in the Yangtze finless porpoise (*Neophocaena asiaeorientalis asiaeorientalis*)

**DOI:** 10.1038/srep22471

**Published:** 2016-03-02

**Authors:** Rui Ruan, Jue Ruan, Xiao-Ling Wan, Yang Zheng, Min-Min Chen, Jin-Song Zheng, Ding Wang

**Affiliations:** 1 Key Laboratory of Aquatic Biodiversity and Conservation of the Chinese Academy of Sciences, Institute of Hydrobiology, Chinese Academy of Sciences, Wuhan 430072, China; 2 The University of Chinese Academy of Sciences, Beijing 100039, China; 3 Agricultural Genomes Institute at Shenzhen, Chinese Academy of Agricultural Sciences, Guangdong 518120, China

## Abstract

Little is known about the major histocompatibility complex (MHC) in the genome of Yangtze finless porpoise (*Neophocaena asiaeorientalis asiaeorientalis*) (YFP) or other cetaceans. In this study, a high-quality YFP bacterial artificial chromosome (BAC) library was constructed. We then determined the organization and characterization of YFP MHC class II region by screening the BAC library, followed by sequencing and assembly of positive BAC clones. The YFP MHC class II region consists of two segregated contigs (218,725 bp and 328,435 bp respectively) that include only eight expressed MHC class II genes, three pseudo MHC genes and twelve non-MHC genes. The YFP has fewer MHC class II genes than ruminants, showing locus reduction in DRB, DQA, DQB, and loss of DY. In addition, phylogenic and evolutionary analyses indicated that the DRB, DQA and DQB genes might have undergone birth-and-death evolution, whereas the DQB gene might have evolved under positive selection in cetaceans. These findings provide an essential foundation for future work, such as estimating MHC genetic variation in the YFP or other cetaceans. This work is the first report on the MHC class II region in cetaceans and offers valuable information for understanding the evolution of MHC genome in cetaceans.

The major histocompatibility complex (MHC) plays a vital role in the vertebrate immune system, recognizing and presenting pathogen-derived peptides to receptors on CD8+ and CD4+ T lymphocytes^1^. MHC genes are mainly classified as class I and class II genes. The class I genes are primarily involved in immune defense against intracellular pathogens, whereas the class II genes are predominantly responsible for monitoring the extracellular environment for foreign peptides^2^. The MHC genomic structure of some mammals (e.g., human, rat, dog, cat, horse, pig, sheep and cattle), particularly the MHC class II regions, have been studied extensively^3^,^4^,^5^,^6^,^7^,^8^,^9^. This region displays highly conserved organization in a wide range of mammals^10^, and MHC class II genes usually exhibit high intraspecific diversity, possibly reflecting the need to respond to foreign peptides derived from parasites^11^,^12^.

However, little is known about the content and organization of the MHC class II region in cetaceans. Sequence variation and polymorphism analyses of exon 2 of DRB and DQB have been performed for some cetacean species^13^,^14^,^15^,^16^,^17^,^18^,^19^. Though, studies about the expression of DRB and DQB genes have been reported in a few cetaceans^20^,^21^,^22^, the expression and functionality of most MHC class II genes has not been considered in most studies^14^,^16^,^17^,^23^,^24^, and gene duplication must be confirmed. For example, only a single DQB locus had been reported in finless porpoises (*Neophocaena phocaenoides*) inhabiting Japanese waters^18^ and in some other toothed whales^15^,^19^,^20^. By contrast, multiple DQB loci have been described in the finless porpoise populations living in Chinese waters^23^,^25^, which were reclassified in 2009 as two different species: the Indo-Pacific finless porpoise (*N. phocaenoides*) and the narrow-ridged finless porpoise (*N. asiaeorientalis*)^26^,^27^,^28^.

The Yangtze finless porpoise (*N. a. asiaeorientalis*) (YFP) is the sole freshwater subspecies of *N. asiaeorientalis* and is found in the middle and lower reaches of the Yangtze River and the adjoining Poyang and Dongting lakes^29^,^30^. Due to its small population size, sharply declining population, and high probability of extinction, the YFP was recently reclassified as a critically endangered population on the IUCN (International Union for Conservation of Nature) Red List^29^,^30^. In most mammals, MHC loci are highly variable, which may be an adaptive strategy against the large number of pathogens encountered by natural populations^13^,^31^. Thus, species or populations with a low level of MHC diversity might be particularly vulnerable to infectious diseases, leading to a decline of population viability^32^. A previous study revealed abundant genetic variations in DQB exon 2 and suggested that the YFP population had a much higher level of DQB diversity than marine populations^23^. However, such estimation of MHC diversity for this population may be inaccurate, because multiple DQB loci might have been amplified simultaneously and been analyzed as a single DQB locus, besides only a single MHC class II gene was analyzed in this study, which could not well reflect genetic variations of MHC genes in YFP^23^. Thus, determining the organization and characterization of the YFP MHC class II region will provide an essential foundation for estimating MHC genetic diversity and resistance/susceptibility to pathogens, exploring the mechanism of mate choice, and other conservation genetics studies based on MHC markers in the YFP. In addition, during the evolutionary transition from land to a marine environment approximately 50 million years ago, cetaceans have undergone numerous critical challenges^33^,^34^, and their immune system genes (such as MHC genes) may reflect this shift in habitat. Thus, characterizing the YFP MHC class II region is also of significant importance for exploring adaptive evolution in the cetacean MHC.

In this study, a high-quality bacterial artificial chromosome (BAC) library was constructed for the YFP for *in vitro* genetic resource conservation and to characterize the YFP MHC genomic structure. We investigated the genomic organization of the YFP MHC class II region in this work and conducted comparative and phylogenic analyses of the MHC class II genes in this region. Determining the organization and characteristics of the YFP MHC class II genes will provide a solid foundation for evaluating MHC genetic diversity in future work and support valid evolutionary inferences about non-model species such as cetaceans. In addition, this work provides the first genomic sequence map of MHC class II region in Cetacea and may be a valuable resource for further cetacean MHC genomic research.

## Results

### YFP BAC library construction

A BAC library including approximately 440,000 clones was successfully constructed for the YFP with an average insert size of approximately 113 kb and an empty vector rate of approximate 5.4% (Fig. S1). The library contained 14.8-fold genome equivalents based on the YFP genome size of 3.17 × 10^9 ^ bp^35^. This library thus has applications in *in vitro* conservation of genetic resources and as a tool for MHC genomic research.

### Sequencing and assembly of the YFP MHC class II contigs

**Figure 1:**
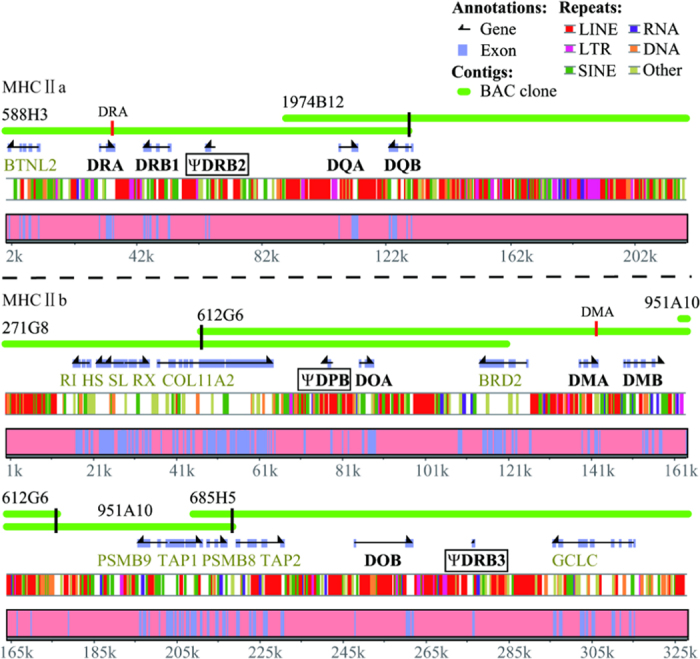
Genomic structure and content of the Yangtze finless porpoise MHC class II region, which was divided into two segregated subregions (MHC IIa and MHC IIb). Black boxes suggest pseudogenes. RI indicates RING1; HS indicates HSD17B18; SL indicates SLC39A7; RX indicates RXRB; ψ indicates pseudogene. The black bold genes indicate MHC class II genes in the Yangtze finless porpoise MHC class II region. The light green genes represent non-MHC genes in the Yangtze finless porpoise MHC class II region. The red vertical bars in BAC clones represent the binding sites for DRA and DMA primers of screening library, the black vertical bars representing the binding sites for end-primers of screening library.

Two BAC clone-based contigs bearing MHC class II genes were identified, consisting of two (588H3 and 1974B12) and four (271G8, 612G6, 951A10 and 685H5) overlapping BAC clones, respectively (Fig. 1). These six clones were sequenced using the Illumina Hiseq 2000 platform. One billion bases were generated from each BAC clone, which were then assembled and aligned into two consensus sequences of 218,725 bp and 328,435 bp without any gap (Fig. 1, Table 1). These six BAC clone sequences and the two consensus sequences have been deposited into GenBank with accession numbers KP114539 – KP114544 and KT804703 – KT804704. In this study, the YFP MHC was designated as MhcNeas (in which Neas is composed of the first two letters of the genus name *Neophocaena* and the first two letters of the species name *asiaeorientalis*) according to the proposal for MHC nomenclature^36^.

### Identification of the YFP MHC class II genes

After repeat masking, gene prediction was conducted using the Genscan and Fgenesh programs and BLAST alignment. A total of 23 genes were identified in the MHC class II region of the YFP. The contig sequence of 218,725 bp included six genes (BTNL2, **DRA**, **DRB1**, **ψDRB2**, **DQA** and **DQB**), and 17 genes were detected in the other contig sequence of 328,435 bp, including RING1, HSD17B8, SLC39A7, RXRB, COL11A2, **ψDPB**, **DOA**, BRD2, **DMA**, **DMB**, PSMB9, TAP1, PSMB8, TAP2, **DOB**, **ψDRB3** and GCLC (the bold text indicates YFP MHC class II genes, the others are non-MHC genes) (Fig. 1). Eight MHC class II genes (DRA, DRB1, DQA, DQB, DMA, DMB, DOA and DOB) were further verified as expressed genes by cDNA PCR. The mRNA sequences of these eight genes have been submitted to GenBank with accession numbers KP114553 – KP114560.

### Comparative genomics analysis of the YFP MHC class II region

**Figure 2:**
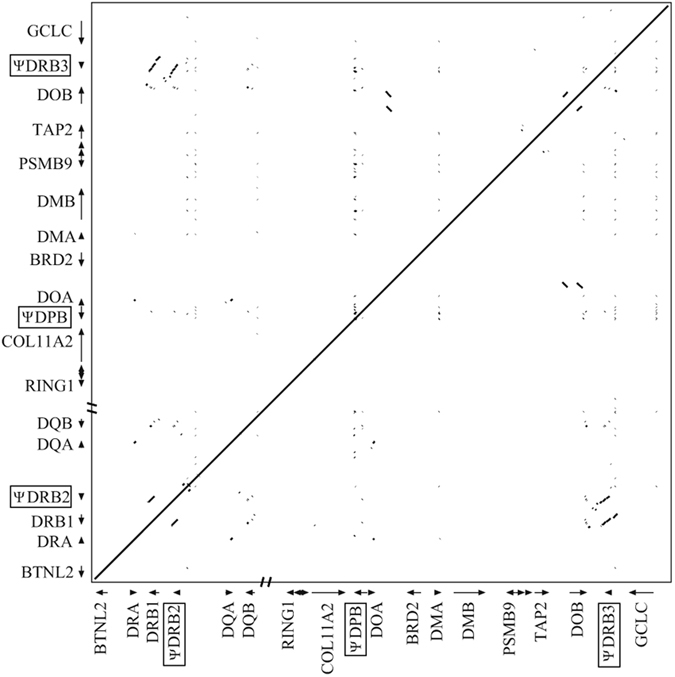
Dot matrix analysis of the Yangtze finless porpoise MHC class II nucleotide sequences itself. The dot plot was performed using Pipmaker.

**Figure 3:**
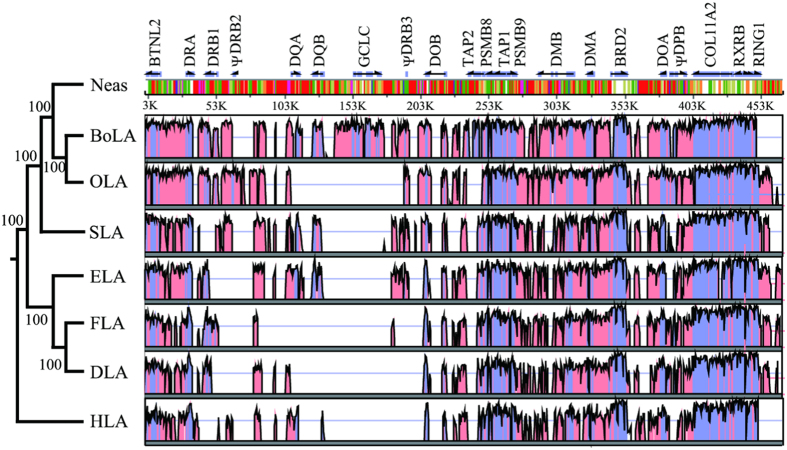
Phylogenetic relationship and VISTA plot of the MHC class II nucleotide sequences from eight mammal species: human (*Homo sapiens*), cattle (*Bos taurus*), sheep (*Ovis aries*), pig (*Sus scrofa*), horse (*Equus caballus*), dog (*Canis familiaris*), cat (*Felis catus*) and Yangtze finless porpoise (*Neophocaena asiaeorientalis asiaeorientalis*). The corresponding MHC class II regions are labeled HLA, BoLA, OLA, SLA, ELA, DLA, FLA and Neas, respectively. For the sake of comparative sequence analysis, the orientations of the MHC class IIb regions from Yangtze finless porpoise, cattle and sheep were inverted. The height of the bars represents the repeats distributing in Neas. Different color bar represents different kind of repeat as that showed on Fig. 1.

The dot-plot analysis of the YFP MHC class II region itself showed three intra-MHC repeats corresponding to DRB1, ψDRB2 and ψDRB3 regions (Fig. 2). Additionally, another three relatively small repeat regions were yielded by the self-dot plot of Neas, which were probably specific repeat sequences in Yangtze finless porpoise MHC region (Fig. 2). In addition, the genomic organizations of the MHC class II regions of YFP (Neas), human (HLA), cattle (BoLA), sheep (OLA), horse (ELA), dog (DLA) and cat (FLA) were compared using VISTA with the LAGAN alignment program. The VISTA plot revealed two highly variable segments in the mammalian MHC II regions, namely the DRB1-DQB and GCLC-ψDRB3 subregions (Fig. 3).

**Figure 4:**
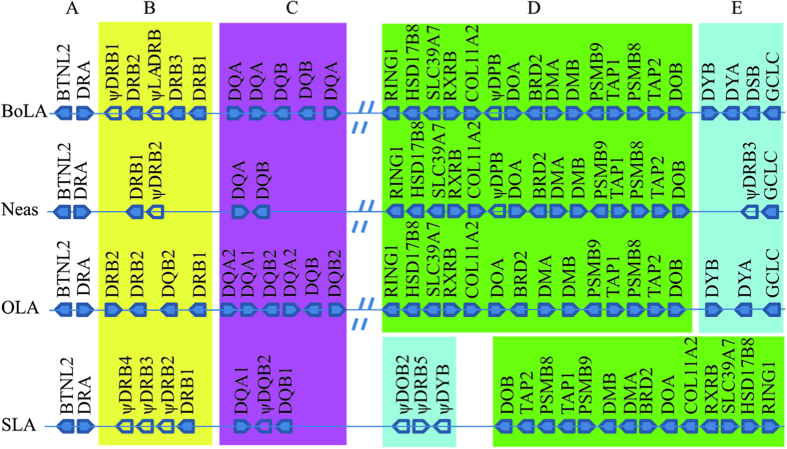
Schematic comparison of the MHC class II region organization of cattle (BoLA), sheep (OLA), pig (SLA) and Yangtze finless porpoise (Neas). Different colors indicate different categories: white, (**A**); yellow, (**B**); purple, (**C**); green, (**D**); and blue, (**E**). Dotted lines indicate breakpoints. Unfilled boxes and ψ indicate pseudogenes. The structure of the BoLA class II region is depicted according to the genome sequence of *Bos taurus* chromosome 23 (NCBI Reference Sequence: AC_000180.1), the structure of the OLA referring to Gao *et al*.^9^, and the structure of the SLA class II region according to the genomic sequence of SLA^7^.

To explore the changes in the two highly variable segments in YFP after the separation of Cetacea and Artiodactyla, MHC class II gene numbers and their orders in Neas, BoLA, OLA and SLA were compared (Fig. 4). And based on the locus distribution of MHC class II genes, the MHC class II regions among the four species could be divided into five parts, including two conserved segments (A and D) that contain stable number of genes, and three highly variable segments (B, C and E) that contain different number of MHC class II genes (Fig. 4). A and D corresponded to BTNL2-DRA and DOB-RING1, respectively, while B, C and E corresponded to the DRB, DQ and DY subregions, respectively (Fig. 4). In the highly variable subregions (B and C), the major differences among the four species were the numbers of DRB and DQ loci. Because DSB evolved from a DRB-like sequence^5^, and thus, in the highly variable E subregion, DRB is expressed in BoLA but it is a pseudogene in both Neas and SLA. The DY genes (DYA and DYB) have been lost in Neas. By comparison, the loci of MHC class II genes have decreased in the YFP (Fig. 4).

### Evolutionary process and selection pressure analyses

**Figure 5:**
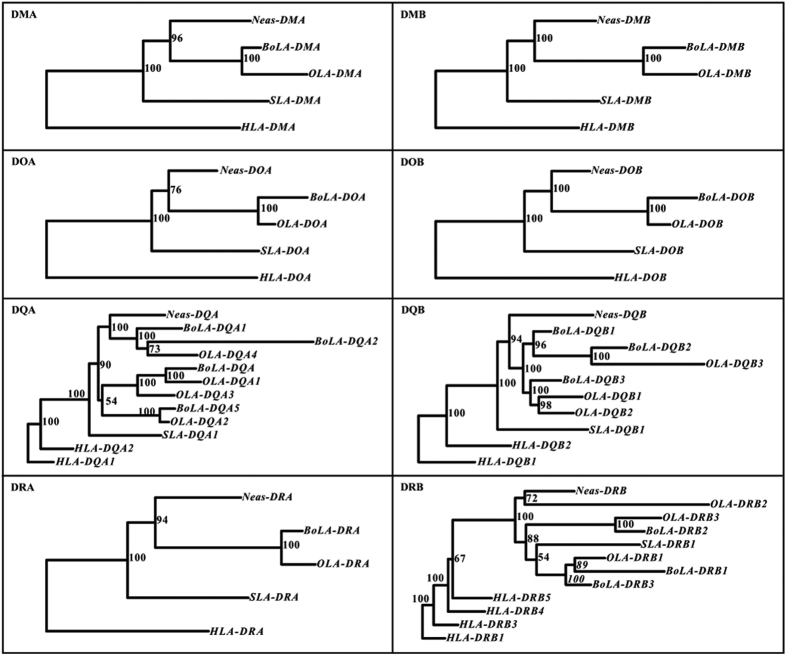
Phylogenetic trees based on the coding sequences of eight MHC class II genes from cattle (BoLA), sheep (OLA), pig (SLA) and Yangtze finless porpoise (Neas). MrBayes and PhyML software yielded phylogenetic trees with similar topologies, and thus only Bayesian trees are provided. Numbers near nodes indicate Bayesian posterior probabilities.

Phylogenetic trees were constructed to explore the evolutionary process of the YFP or cetacean MHC class II genes. First, we constructed a phylogenetic tree based on the entire sequence of the MHC class II region in Neas, BoLA, OLA, SLA, ELA, FLA, DLA and HLA (Fig. 3). This tree revealed that the order of species divergence is consistent with the phylogenetic analyses based on other nucleotide sequences, supporting Cetacea is closer to Ruminantia (cattle and sheep) than Suidae (pig)^37^,^38^. The birth-and-death process is a very important model for the evolution of mammalian MHC class II genes^10^,^39^. Thus, to further explore the evolutionary history of the YFP MHC class II genes, phylogenetic trees were also constructed using the coding region sequences (CDSs) of DRA, DRB, DQA, DQB, DMA, DMB, DOA and DOB from YFP, cattle, sheep and pig, with human as the outgroup (Fig. 5). The phylogenetic relationships of the eight genes were not consistent. The phylogeny of CDSs for DRA, DMA, DMB, DOA and DOB shared the same tree topology as the tree constructed using the entire MHC class II region (Fig. 3), whereas the phylogenetic trees for the CDSs of DRB, DQA and DQB had quite different tree topologies (Fig. 5).

When evolutionary selection analyses were conducted on the CDSs of the eight MHC class II loci (DRA, DRB, DQA, DQB, DMA, DMB, DOA and DOB) in mammals (human, cattle, sheep, pig and seven cetacean species) (Table S5), all d_N_/d_S_ ratios were less than 1 (Table 2). However, when evolutionary selection analysis was conducted on the eight MHC class II loci in the seven cetacean species, the ratio of d_N_/d_S_ was significantly larger than 1 for the DQB locus (d_N_/d_S_ = 2.125, *P* = 6.36E-4) (Table 2), indicating positive selection at the DQB locus. In addition, MHC class II genes are classified into classical MHC class II genes (DR, DQ and DP) and non-classical MHC class II genes (DM and DO). The non-classical MHC class II proteins’ roles are known in antigen-presenting cells, which do not directly bind antigens^40^. However, the classical MHC class II proteins bind antigens with peptide-binding region, which is mainly coded by the second exon^41^. And thus, the d_N_/d_S_ ratios of exon 2 and the rest CDS (except exon 2) of classical MHC class II genes (DR and DQ) were respectively estimated in mammals and in cetaceans to explore which part is mainly subjected to positive selection. The result also showed that only the d_N_/d_S_ of exon 2 of DQB in cetaceans is significantly larger than 1 (Table S6). YFP inhabits in the freshwater environment, which is quite different from the marine environment. And thus, to eliminate the possible effect from YFP, the d_N_/d_S_ ratios of exon 2 of DQB was further estimated only in marine cetaceans (excluding YFP), which is also significantly larger than 1 (d_N_/d_S_ = 2.58, *P* = 3.75E-4).

## Discussion

The population genetic structure of the YFP, as revealed by mitochondrial and microsatellite DNA analyses, indicates that the YFP is becoming a genetically fragmented population and emphasizes the need for genetic conservation of this critically endangered population^42^. Thus, the genetic diversity of the YFP must be further investigated, particularly regarding the variability of MHC genes, to evaluate its long-term survival probability. However, little is known about the MHC genomic structure of the YFP or any other cetacean species, limiting the study of MHC genetic diversity. Therefore, in this work, we first constructed a high-quality BAC library for the YFP and then determined the organization and characteristics of the MHC class II genes.

Based on the high-quality YFP BAC library we constructed, two contigs (MHC IIa and IIb) bearing the YFP MHC class II genes were mapped, sequenced, assembled and annotated (Fig. 1). According to the arrangement of MHC class II genes in YFP (Fig. 4), it is very possible that the MHC class II region has been also interrupted and divided into two segregated subregions (MHC IIa and MHC IIb) in Cetacea like ruminants^9^,^43^; in cattle and sheep there are approximate 18.5 Mb between these two segregated suregions^9^. There are three evidences for this inference. First, a sequence of approximately 80 kb after the DQB locus did not contain any MHC class II gene, and there was an overlapping fragment (approximately 40 kb) between BAC clones 588H3 and 1974B12. This indicates that the YFP MHC class IIa region obtained in this study was complete and authentic. Second, the location of the GCLC (glutamate-cysteine ligase, catalytic subunit) gene is far from the MHC class II region in those mammals (e.g., human, dog, cat, horse and pig), of which the genomes were well sequenced and studied. For example, according to available genome data in NCBI, the distance is approximately 20 Mb in human, dog, cat and horse and approximately 2 Mb in pig. However, the GCLC gene is very close to the MHC class II region in the YFP, cattle^5^ and sheep^9^ (Fig. 4). The results also suggested that the structure of the YFP MHC class II region is similar to those in cattle and sheep. Third, genome data in NCBI indicate that the MHC class II region in killer whale (*Orcinus orca*) (NW_004438672, NW_004438437) is also interrupted by other sequences. Taken together, these support the inference that the YFP MHC class II region might be interrupted into two segregated subregions, possibly as the hypothesis in BoLA and OLA that a large inversion of the ancestral chromosome has disrupted the ancestral MHC class II region into IIa and IIb sub-regions^9^. However, the reported swine MHC class II region is not interrupted by other DNA fragments^7^. From the phylogenetic trees constructed using the whole MHC class II sequences (Fig. 3), we can infer that the breakpoint in the MHC class II region might have occurred after the divergence of swine and Cetartiodactyla but before the divergence of Cetacea and ruminants.

When gene prediction was conducted on the MHC class II region of the YFP, only 11 MHC class II genes were identified (DRA, DRB1, ψDRB2, DQA and DQB in MHC IIa; ψDPB, DOA, DMA, DMB, DOB and ψDRB3 in MHC IIb) (Fig. 1). Of these, only eight MHC class II genes (DRA, DRB1, DQA, DQB, DMA, DMB, DOA and DOB) were further verified as expressed genes by cDNA PCR. Comparative analyses of this MHC class II region clearly indicated that the YFP has fewer MHC class II loci than cattle and sheep, particularly in the three highly variable segments (B, C, E) (Fig. 4).

To explore the deleting process of MHC class II loci in the YFP, the evolutionary history of the eight expressed MHC class II genes (DRA, DRB1, DQA, DQB, DMA, DMB, DOA, DOB) after the divergence of Cetacea and ruminants was investigated through comparative analyses of their phylogenetic trees constructed for Neas, BoLA, OLA and SLA, using HLA as the outgroup. The phylogenetic trees for the DRA, DOB, DOA, DMB and DMA genes showed similar topology (Fig. 5). By contrast, phylogenetic analysis of the DRB, DQA and DQB genes indicated that the sequences might be clustered according to gene categories (e.g. orthologous or paralogous genes) than species (Fig. 5). But the tree topologies among them were not similar, which may reflect divergent birth-and-death evolution of the DRB, DQA and DQB genes.

In the phylogenetic tree of the DRB genes, DRB genes were clustered into three clades according to gene categories among the YFP, pig, cattle and sheep (Fig. 5). Neas-DRB and OLA-DRB2 were clustered into a clade that was older than the other two clades. SLA-DRB1 was clustered with BoLA-DRB3, BoLA-DRB1 and OLA-DRB1 into a younger clade. However, from the phylogenetic relationship showed in Fig. 3, we can see that SLA was an older clade than cetaceans and ruminants. And thus, based on the three clades showed in Fig. 5, we could infer that at least three DRB loci were present in their ancestral MHC class II region before the divergence of Cetacea and ruminants, but as shown in the Neas B subregion of Fig. 4, at least one DRB locus was entirely deleted in the evolutionary process.

The phylogenetic tree of the DQA genes revealed that the DQA genes were also grouped according to gene categories among the YFP, cattle and sheep, producing two clades (Fig. 5). Thus, there were at least two DQA loci before the divergence of Cetacea and ruminants, and the YFP lost one DQA locus thereafter (Fig. 4). The phylogenetic tree of the DQB genes revealed that DQB genes were clustered into two groups after the divergence of Cetacea and ruminants. The DQB phylogenetic tree includes only one DQB locus in the YFP, but the cetacean and ruminants DQB loci may be paralogous genes, as Yang *et al*. presumed that the sequences of cetaceans and artiodactyls (including ruminants) were paralogous in the DQB genes^44^. Yang *et al*. hypothesized that gene-duplication events occurred at least once in the DQB genes prior to the cetacean/artiodactyl divergence^44^. Gene duplication could give rise to two sets of paralogous genes (the A set and B set). The A sets of all artiodactyls have survived to the present day, whereas the B sets were lost or became pseudogenes. By contrast, the B sets in cetaceans were preserved, whereas the A sets in cetaceans were lost^44^. As shown in the C subregion in Fig. 4, at least one DQB locus and one DQA locus have been lost since the cetacean/ruminants (BoLA and OLA) divergence.

In comparison with the BoLA II and OLA II genes, the YFP has fewer DRB, DQA and DQB loci in the MHC class II region. Thus, this region underwent the birth-and-death evolutionary process, and at least one locus might have been lost among the three genes in the evolutionary process of cetaceans. The DYA and DYB genes were also lost in Neas (Fig. 4). Based on the phylogenetic tree of the eight species (Fig. 3), we inferred that DY genes were lost in cetaceans after the divergence of Cetacea and ruminants. These changes in cetacean MHC regions might be related to their ancestral shift in habitat from land to a marine environment. Due to the lower prevalence of infectious diseases in the marine environment than the land environment, these genes, which play an essential role in defense against foreign peptides derived from parasites, might have been under less evolutionary selection pressure^45^.

The ratio of d_N_/d_S_ indicates the selection pressure under which the coding sequence evolved: d_N_/d_S_ > 1 is interpreted as signifying positive selection, whereas d_N_/d_S_ < 1 indicates purifying selection^46^. Based on Table 2 and Table S6, we determined that the eight expressed MHC II genes in mammals have evolved under purifying selection, with the exception of the DQB locus in cetaceans. In cetaceans, the DQB subjected to positive selection mainly focuses on the exon 2, which showed the importance of the exon 2 as peptide-binding region. The evolutionary selection analyses suggested that the eight expressed MHC class II genes in cetaceans might have evolved under purifying selection when the ancestor of cetaceans shifted from land to a marine environment; however, after the cetaceans fully adapted to aquatic life, the DQB locus have evolved under positive selection to defend against different marine pathogens encountered during the process of speciation or population expansion. This result is consistent with a previous study suggested positive selection pressure on the exon 2 of DQB in bottlenose dolphin^19^, further indicating that the DQB locus may play a key role in the cetacean evolutionary process. Thus, sequence variation at the exon 2 of DQB locus should be examined more closely in future evaluations of genetic variability in the YFP or any other cetacean species.

In this work, only one DQB locus was identified in the YFP, whereas Xu *et al*. reported that at least three copies of the DQB gene are present in the finless porpoise^25^. However, a single DQB locus in the YFP is consistent the presence of a single DQB locus in many toothed whales^15^,^19^,^20^. Although there are three DRB genes in the YFP MHC class II region (Fig. 1), only the DRB1 gene is functional, whereas ΨDRB2 has lost exon 1, a part of exon 2 and exon 6 (Fig. S2) and ψDRB3 has retained only exon 3. Exon 2 of the DRB locus has typically been investigated in previous studies of DRB genetic variability^14^,^17^,^24^. Because exon 2 of ψDRB2 in YFP has only lost the first fourteen bases (Fig. S2), inappropriate primer design could lead to the amplification of this exon 2, and consequently, result in overestimation of DRB genetic variability in the YFP. Given the critically endangered status of the YFP, reevaluation of the population genetic diversity of MHC class II genes is urgently needed. Because the DQB locus is under positive selection in cetaceans, assessing the genetic variability of the DQB locus in the YFP population is particularly important. In addition, most studies investigating MHC variability have focused on a single or very few MHC loci due to the lack of valid MHC markers, hindering the elucidation of the intrinsic molecular mechanism^12^. Thus, this exploration of organization and characteristics of the YFP MHC class II region provides an essential foundation for future work, such as estimating MHC genetic variation in the YFP. This work is also the first report on the MHC class II region in cetaceans and offers valuable information for understanding the evolution of the MHC genome in cetaceans.

## Materials and Methods

### Tissue collection

To provide high quality genomic DNA for BAC library construction in this study, liver tissue sample was collected from a female YFP that was accidentally killed by a fishing boat in the main stream of the Yangtze River. In addition, to obtain mRNA to verify the functionality of the MHC class II gene identified in this study, blood sample was drawn from the vein in the fluke of a captive YFP at Wuhan Baiji Dolphinarium by using one-off syringe. Necropsy and sampling were conducted systematically in accordance with all ethical guidelines and legal requirements in China. The protocol of this study was approved by the Institutional Review Board of the Institute of Hydrobiology, Chinese Academy of Sciences.

### BAC library construction and positive clone screening

Liver tissue was ground with liquid nitrogen and resuspended in ice-cold phosphate-buffered saline. High-molecular-weight DNA (120–170 kb) was extracted following the protocol described by Osoegawa^47^ and Zeng^48^. A BAC library was constructed for this YFP using the Copy Control BAC Cloning Kit and TransforMax EPI300 Electrocompetent *E. coli* according to the manufacturer’s protocol (Epicentre, Madison, USA) and a previously reported protocol for BAC library construction^47^. To conserve space, each two BAC clones were preserved in one well of a 96-well plate. The BAC library consisted of approximately 440,000 clones and was preserved in 2,290 96-well plates.

Previous studies suggested that the MHC class II regions have a conservation of synteny among certain mammals^7^,^8^,^10^, so we employed a walking method to construct the YFP MHC class II contig(s) in this study. Library screening was performed using a 4D-PCR method^49^. Firstly, two pairs of PCR primers (DRA and DMA) (Table S1) were used to screen the BAC library. To confirm positive clones containing amplicons of DRA and DMA, the amplified products were cloned and sequenced, and then identified by BLAST sequences in NCBI database. And then, end-sequence all real positive BAC clones, primer design of their end sequences and amplifying these positive BAC clones were performed according to reported method^48^. The positive BAC clone, which had the longest insert size and also had effective end-primer pair at both ends, would be chosen as the best positive BAC clone for the two primer pairs (DRA and DMA) and also as the starting point of next step. Secondly, library screening, positive clone end-sequencing and primer design were repeated to construct the contig until boundaries of MHC class II region were included in certain BAC clones. All the primer pairs that had been used to screen positive clones and to construct the contigs of YFP MHC class II region were listed in Table S1.

### Positive BAC clones sequencing and assembly

DNA was extracted from each positive BAC clone using the AxyPrep^TM^ Plasmid Kit (Axygen Biosciences, China) to avoid *E. coli* genomic contamination. The extracted DNA was sent to Shanghai Majorbio Bio-pharm Technology Company for high-throughput sequencing on the Illumina Hiseq 2000 platform. Clean reads were obtained by removing reads containing adapter sequences, poly-N reads and low-quality reads (containing >50% bases with sequence quality <15) from the raw reads. The clean reads were assembled using SOAPdenovo software^50^. A primer-walking method was applied to fill any gaps in the assembled sequences.

### Gene identification and verification

The complete sequences of BAC clones were aligned into two consensus sequences using Blast2Seq^51^. Two full-length sequences of the MHC class II region were analyzed by RepeatMasker in a Linux operating system, with Cetacea as the organism-specific parameter. Genes were identified using two different gene prediction programs: Genscan and Fgenesh^52^,^53^. In the Genscan and Fgenesh algorithms, the organism-specific parameters were set as vertebrate and cow, respectively. The predicted gene and protein sequences were aligned with NCBI reference RNA sequences using BLASTN^54^ and non-redundant protein sequences with BLASTP^55^, respectively, to identify the boundaries of exons and expressed genes or pseudogenes.

To further verify the functionality of the predicted MHC class II genes, cDNA PCR was performed. Firstly, mRNA was extracted from the blood sample of a captive YFP by using RNAprep Pure Kit (TIANGEN, Beijing, China). And then, after quality check, mRNA was reversely transcribed into cDNA by using RevertAid First Strand cDNA Synthesis Kit (Thermo Scientific, made in EU). Finally, the obtained cDNA was used as template to conduct PCR amplification. At the same time, based on the YFP MHC class II region sequence obtained in this study and those homologous sequences of closely related species downloaded from GenBank, eight primer pairs were designed for the eight MHC class II genes by using Primer Premier 5.0 (Table S2). The PCR products were cloned, and two clones from each primer were chosen to sequence according to previously reported methods^56^.

### Comparative genomics analysis

The self-dot matrix of the YFP MHC class II region sequence was created using PipMaker^57^. For comparative analysis, we downloaded the homologous MHC class II region sequences of human (*Homo sapiens*), cattle (*Bos taurus*), sheep (*Ovis aries*), pig (*Sus scrofa*), horse (*Equus caballus*), dog (*Canis familiaris*) and cat (*Felis catus*) from GenBank (Table S3). The annotated MHC class II region sequence of the YFP and the trimmed corresponding sequences of mammals from previous reports were aligned using VISTA^58^ with the LAGAN alignment program. LAGAN is a system for global pair-wise and multiple alignment of genomic sequences and may be more suitable than other pairwise alignments for aligning conserved exons between distant species^59^.

### Evolutionary process and selection pressure analyses

To construct the phylogenetic tree, the MHC class II region sequences of the YFP and the aforementioned seven mammals were aligned using the programs Mauve 2.3.1^60^ and MAFFT (version 7)^61^ and checked manually. The molecular evolution model of the sequences was generated by MrModeltest 2.3^62^, and the phylogenetic tree was constructed by MrBayes 3.2.2^63^ with 1,000,000 generations.

To explore the evolutionary history of the eight YFP MHC II genes, the coding sequences of the MHC II genes except DMB were completely amplified by using the eight primers listed in Table S2 and the cDNA from a captive YFP, and sequenced. The corresponding sequences of human, cattle, sheep and pig were downloaded from GenBank (Table S4). In this process, we collected as many expressed or predicted genes as possible to construct phylogenetic trees. The coding sequences were also aligned using MAFFT software. Phylogenetic trees were constructed using the protocol described above for the entire MHC II gene sequences. In addition, another phylogenetic tree was constructed using the software PhyML (version 3.1) with 1000 bootstrapping replicates^64^. The molecular evolution model of the nucleotide sequence was estimated by Modeltest 3.7^65^.

Because the ratio of non-synonymous and synonymous substitutions (d_N_/d_S_) indicates the selection pressure under which the coding sequence evolved, d_N_/d_S_ was calculated in MEGA5.0, and the Z-test statistical method was performed^66^. To strengthen the reliability, before calculating d_N_/d_S_, the substitution saturation was measured using DAMBE (version 5.3.108)^67^. The overall d_N_/d_S_ ratios of the eight MHC class II genes CDS in mammals and in cetaceans were estimated (Table S5). In addition, due to the exon 2 of classical MHC class II genes (DRA, DRB, DQA and DQB) mainly involving in the peptide-binding region, the d_N_/d_S_ ratios for the exon 2 and the rest CDS (excluding exon2) of the four classical MHC class II genes were also estimated in mammals and in cetaceans to verify the results obtained based on CDS.

## Additional Information

**How to cite this article**: Ruan, R. *et al*. Organization and characteristics of the major histocompatibility complex class II region in the Yangtze finless porpoise (*Neophocaena asiaeorientalis asiaeorientalis*). *Sci. Rep*. **6**, 22471; doi: 10.1038/srep22471 (2016).

## Supplementary Material

Supplementary Information

## Figures and Tables

**Table 1:** Information on the six BAC clones covering the Yangtze finless porpoise MHC class II region.

BAC clone ID	GenBankaccession number	Insertlength (bp)	GC%	MHC IIregion
588H3	KP114539	129785	48.4	a
1974B12	KP114540	128717	47.9	a
271G8	KP114541	120854	50.1	b
612G6	KP114542	129597	50.4	b
951A10	KP114543	59281	51.2	b
685H5	KP114544	119211	52.0	b

**Table 2:** d_N_/d_S_ ratios calculated for eight MHC class II genes in mammals (human, cattle, sheep, pig and seven cetacean species) and in cetaceans (seven cetacean species: the Yangtze finless porpoise, common bottlenose dolphin, Indo-Pacific bottlenose dolphin, killer whale, Yangtze River dolphin, sperm whale, and minke whale).

Loci	Mammals		Cetaceans	
	d_N_/d_S_	*P*-Value	d_N_/d_S_	*P*-Value
DQA	0.504	4.40E-8	0.556	0.022
DQB	0.640	6.08E-4	**2.125**	**6.36E-4**
DRA	0.497	5.13E-6	0.510	0.015
DRB	0.585	2.52E-4	0.979	0.500
DMA	0.406	2.45E-9	0.660	0.055
DMB	0.546	8.43E-5	1.407	0.133
DOA	0.418	1.90E-8	0.567	0.038
DOB	0.407	9.42E-8	0.396	1.86E-3

Detailed sample information is provided in Table S5. The bold text indicates that the d_N_/d_S_ ratio was significantly greater than 1.
