# Supplementary material for: Organization and characteristics of the major histocompatibility complex class II region in the Yangtze finless porpoise (Neophocaena asiaeorientalis asiaeorientalis)
Source: Sci Rep. 2016 Mar 2;6:22471. doi: 10.1038/srep22471 (PMC4773811; doi:10.1038/srep22471)
Supplement: Supplementary Information [file srep22471-s1.doc]

**Organization and characteristics of the major histocompatibility complex class II region in the Yangtze finless porpoise (*Neophocaena asiaeorientalis asiaeorientalis*)**

**Rui Ruan^1,2^, Jue Ruan^3^, Xiao-Ling Wan****^1,2^, Yang Zheng^1,2^, Min-Min Chen^1^, Jin-Song Zheng^1^,*, and Ding Wang^1^,***

^1^ Key Laboratory of Aquatic Biodiversity and Conservation of the Chinese Academy of Sciences; Institute of Hydrobiology, Chinese Academy of Sciences, Wuhan 430072, China;

^2^ the University of Chinese Academy of Sciences, Beijing 100039, China;

^3^ Agricultural Genomes Institute at Shenzhen, Chinese Academy of Agricultural Sciences, Guangdong 518120, China

***Correspondence to:**

Ding Wang (D.W.) or Jin-Song Zheng (J.S.Z.)

Fax: 86-27-87491267

Emails: [wangd@ihb.ac.cn](mailto:wangd@ihb.ac.cn) (D.W.); [zhengjinsong@ihb.ac.cn](mailto:zhengjinsong@ihb.ac.cn) (J.S.Z.)

**
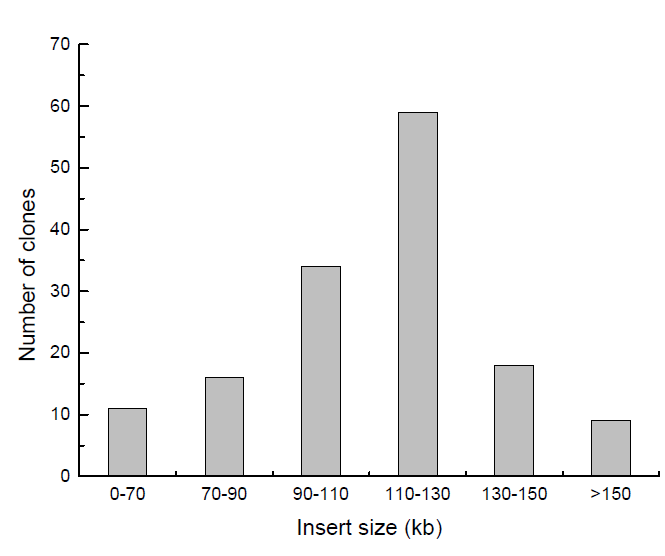
**

**Figure S1.** Distribution of insert sizes of 147 BAC clones in the Yangtze finless porpoise BAC library. Insert sizes were estimated by electrophoresis with BAC-Tracker Supercoiled DNA Ladder (28-165 kb). About of 5.4% non-insert clones were observed in the process of estimating insert size from 147 BAC clones (8 non-insert clones).

**
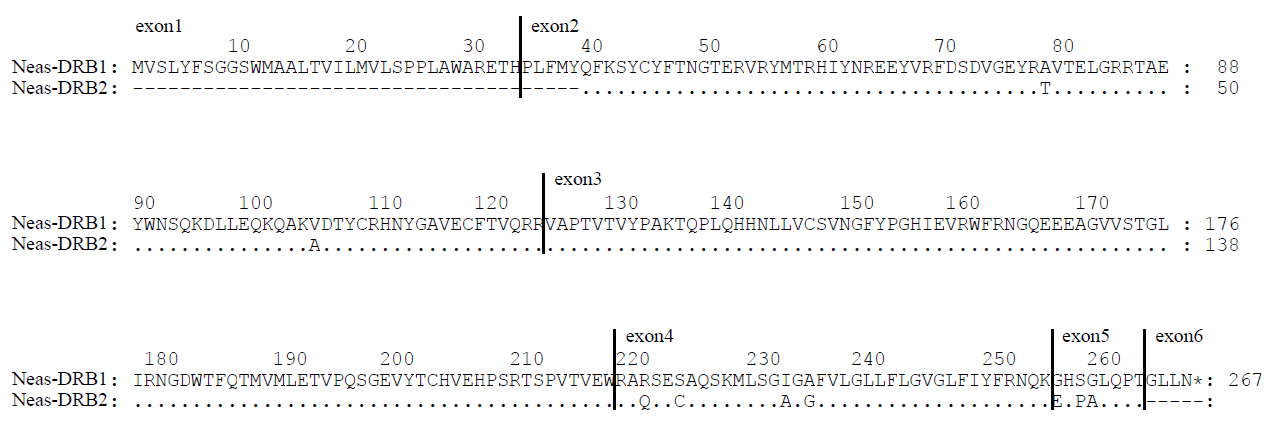
**

**Figure S2.** Predicted amino acid sequence alignment of Neas-DRB1 and Neas-DRB2. Predicted amino acid alignment of Neas-DRB1 and Neas-DRB2 sequences determined in this study. Neas-DRB2 is a pseudogene. Alignment among their predicted protein sequences was generated by MEGA6 and GeneDoc. Dots (.) indicate identity, dashes (-) indicate gaps, asterisk (*) indicates stop codon, and vertical lines (|) indicate exon boundaries.

**Table S1.** Primers used for screening the BAC library to construct the contigs of YFP MHC class II region.

| **Locus** | **Size of amplifications (bp)** | **Primer sequences (5’→3’)** | **Reference** |
| --- | --- | --- | --- |
| DRA | 189 | AATCATGTGATCCAAGCTGAGTTC  TGTTTGGGGTGTTGTTGGAGCG |  |
| DMA | 442 | GGACTGCCCATGAAGGTA  GGAACTAAGGACCAATAAAG | This study |
| 588H3-T7 | 604 | TTGAGGATGGATGGGATT  TGAGCCTGAGAACCGAGT | This study |
| 612G6-T7 | 387 | CAGTATGAATGCCTAATCTC  CTCCACCTCAACAAACAC | This study |
| 612G6-RP1 | 534 | CCCCAAGCATAGGTGAGA  ATGCCAGGAAGTCCACTC | This study |
| 951A10-T7 | 791 | CCCAGATACCCAAACCAT  TGTCGCCTTCAACATAACAT | This study |

The primers for DMA locus, amplifying 442 bp including part of the first intron, were designed based on the DMA DNA sequence of bottlenose dolphin (*Tursiops truncatus*) (GenBank accession number: NW_004207240) by using Primer Premier 5.0. To confirm its effectiveness, the DMA primers were used to perform PCR with the Yangtze finless porpoise genomic DNA as template, and then the amplified product was cloned and sequenced, finally the sequence was identified by BLAST in NCBI database.

Xu S, Sun P, Zhou K, Yang G. Sequence variability at three MHC loci of finless porpoises *(Neophocaena phocaenoid*es)*. Immunogeneti*c**s** 59: 581-592 (2007).

**Table S2. PCR primers of eight MHC class II genes used to detect their expression.**

| **Locus name** | **Primer sequences** | **Length**  **(bp)** | **Including complete CDS** | **Annealing temperature**  **(°C)** |
| --- | --- | --- | --- | --- |
| DRA | F：CAATACAGGAAGGAGCAA  R：TCTACTGACCCTCATCGA | 1118 | Yes | 51 |
| DRB | F：CTTGCCTGCTCCCCTCAC  R：TTCCGGATTTCCAGGTCA | 1030 | Yes | 55 |
| DQA | F：GTGGAATACGCCAGTGAT  R：GGATGGTCCTGAACAGAG | 1006 | Yes | 53 |
| DQB | F：TACTTCTTCCTTTGTTCTC  R：GGTTACAATGTGCTTCTC | 1113 | Yes | 51 |
| DMA | F：ATGGATCATGAGCTGAGC  R：GGTAGGATGTGAGAAACTTG | 862 | Yes | 53 |
| DMB | F：GGACAGGATTCCCCGTGT  R：TCCGCCAGCTGATCAAAC | 758 | No | 54 |
| DOA | F：TAAAGCACCAGGGGACAA  R：ATCCCATTCAAAGTCAGCAC | 896 | Yes | 53 |
| DOB | F：AATGGGTTCTAGCTGGGTT  R：GGAGGAAGAAGGGCACAA | 1010 | Yes | 53 |

**Table S3**. GenBank accession numbers of the MHC class II sequences from human, cattle, sheep, pig, horse, dog and cat used for comparative genomic analysis.

| **Species name** | **MHC name** | **GenBank accession number** |
| --- | --- | --- |
| Human (*Homo sapiens*) | *HLA II* | NT_007592 |
| Cattle (*Bos* *taurus*) | *BoLA* *IIa*  *BoLA* *IIb* | *BoLA* *IIa*: AC_000180  *BoLA* *IIb*: AY957499 |
| Sheep (*Ovis* *aries*) | *OLA* *IIa*  *OLA* *IIb* | *OLA* *IIa*: NW_004080183  *OLA* *IIb*: FJ985855, FJ985860,FJ985871 |
| Pig (*Sus* *scrofa*) | *SLA* *II* | BX323846, BX088590, BX323833, BX324144, BX640585 |
| Horse (*Equus caballus*) | *ELA II* | NW_001867389 |
| Dog (*Canis* *familiaris*) | *DLA* *II* | AJ630362, AJ630363, AJ630364, AJ630365, AJ630366 |
| Cat (*Felis* *catus*) | *FLA* *II* | EU153401 |

**Table S4**. GenBank accession numbers of the MHC class II genes from cattle, sheep and pig used to construct phylogenetic trees.

| MHC II genes | **Human (*Homo sapiens*)** | **Cattle (*Bos taurus*)** | **Sheep (*Ovis aries*)** | **Pig (Sus scrofa)** |
| --- | --- | --- | --- | --- |
| DRA | NM_019111 | NM_001012677 | NM_001280717 | NM_001113706 |
| DRB | DRB1: NM_001243965  DRB3: NM_022555  DRB4: NM_021983  DRB5: NM_002125 | DRB1: XM_005200976  DRB2: XM_582099  DRB3: NM_001012680 | DRB1: NM_001280698  DRB2: XM_004018737  DRB3: XM_004019248 | DRB1: NM_001113695 |
| DQA | DQA1: NM_002122  DQA2: NM_020056 | DQA: XM_003587692  DQA1: NM_001013601  DQA2: NM_001012681  DQA5: NM_001012675 | DQA1: NM_001159759  DQA2: XM_004018908  DQA3: XM_004018909  DQA4: XM_004018907 | DQA1: NM_001130224 |
| DQB | DQB1: NM_002123  DQB2: NM_001300790 | DQB1: NM_001080923  DQB2: NM_001012676  DQB3: NM_001034668 | DQB1: EU176819  DQB2: EU176819  DQB3: XM_004019247 | DQB1: NM_001113694 |
| DMA | NM_006120 | NM_001012674 | XM_004019167 | NM_001004039 |
| DMB | NM_002118 | NM_001040481 | XM_004018740 | NM_001113707 |
| DOA | NM_002119 | NM_001205920 | XM_004018742 | NM_001185143 |
| DOB | NM_002120 | NM_001013600 | XM_004018738 | NM_001114064 |

Species name

**Table S5.** Sequences of MHC class II genes used to calculate the d_N_/d_S_ ratios.

| **Species** | **DRA** | **DRB** | **DQA** | **DQB** | **DMA** | **DMB** | **DOA** | **DOB** |
| --- | --- | --- | --- | --- | --- | --- | --- | --- |
| Common bottlenose dolphin  (*Tursiops truncates*) | XM_004325137 | EF017817 | XM_004317915 | NM_001280608 | NW_004207240 | XM_004326007 | XM_004328684 | XM_004328309 |
| Indo-Pacific bottlenose dolphin (*Tursiops aduncus*) |  | EF017818 |  | EF017816 |  |  |  |  |
| Killer whale (*Orcinus orca*) | XM_004285623 | XM_004285622 | XM_004285618 | NW_004438672 | XM_004267759 | XM_004267810 | XM_004267755 | XM_004267811 |
| Yangtze River dolphin  (*Lipotes vexillifer*) | XM_007459506 |  | XM_007451935 | NW_006778796 | XM_007460810 | XM_007460809 | XM_007460812 | XM_007460803 |
| Sperm whale  (*Physeter catodon*) | XM_007118596 |  | XM_007123824 | XM_007123823 | NW_006713540 | XM_007107873 | XM_007107866 | XM_007107887 |
| Minke whale  (*Balaenoptera acutorostrata*) | XM_007193888 |  |  |  | XM_007186920 | XM_007186925 | XM_007186913 | NW_006728570 |
| Human  (*Homo sapiens*) | NM_019111 | NM_001243965  NM_022555  NM_021983  NM_002125 | NM_002122  NM_020056 | NM_002123 | NM_006120 | NM_002118 | NM_002119 | NM_002120 |
| Cattle  (*Bos taurus*) | NM_001012677 | NM_001012680 | XM_003587692  NM_001013601  NM_001012675 | NM_001080923  NM_001012676  NM_001034668 | NM_001012674 | NM_001040481 | NM_001205920 | NM_001013600 |
| Sheep  (*Ovis aries*) | NM_001280717 | NM_001280698 | NM_001159759  XM_004018908  XM_004018909  XM_004018907 | EU176819  EU176819 | XM_004019167 | XM_004018740 | XM_004018742 | XM_004018738 |
| Pig  (*Sus scrofa*) | NM_001113706 | NM_001113695 | NM_001130224 | NM_001113694 | NM_001004039 | NM_001113707 | NM_001185143 | NM_001114064 |

**Table S6. d_N_/d_S_ ratios calculated for exon 2 and the rest of classical MHC class II genes in mammals and in cetaceans (seven cetacean species). The mammals include human, cattle, sheep, pig and seven cetacean species. The seven cetacean species include the Yangtze finless porpoise, common bottlenose dolphin, Indo-Pacific bottlenose dolphin, killer whale, Yangtze River dolphin, sperm whale, and Minke whale. Detailed sample information is provided in Table S5. The bold text indicates that the d_N_/d_S_ ratio was significantly greater than 1.**

| Loci | | Mammals | | Cetaceans | |
| --- | --- | --- | --- | --- | --- |
|  |  | d_N_/d_S_ | *P*-Value | d_N_/d_S_ | *P*-Value |
| DQA | Exon 2 | 0.589 | 0.012 | 0.674 | 0.227 |
|  | The rest | 0.415 | 1.74E-7 | 0.5 | 0.05 |
| DQB | Exon 2 | 0.811 | 0.228 | **2.288** | **6.17E-4** |
|  | The rest | 0.428 | 1.61E-5 | 0.9 | 0.401 |
| DRA | Exon 2 | 0.556 | 0.023 | 0.912 | 0.430 |
|  | The rest | 0.474 | 4.72E-5 | 0.4 | 5.57E-3 |
| DRB | Exon 2 | 0.740 | 0.106 | 1.417 | 0.166 |
|  | The rest | 0.478 | 9.54E-5 | 0.387 | 0.042 |
